# How Different Medical Practices Are Associated with Types of Patient Complaints in Russian Clinics

**DOI:** 10.3390/healthcare14081027

**Published:** 2026-04-13

**Authors:** Irina Evgenievna Kalabikhina, Anton Vasilyevich Kolotusha, Vadim Sergeevich Moshkin

**Affiliations:** 1 Population Department, Faculty of Economics, Lomonosov Moscow State University, Moscow 119991, Russia; ikalabikhina@yandex.ru; 2 Department of Information Systems, Ulyanovsk State Technical University, Ulyanovsk 432027, Russia; v.moshkin@ulstu.ru

**Keywords:** patient complaints, classification of medical specialties, machine learning in healthcare, organizational aspects of treatment, quality of medical care, Russian healthcare, big data, text analysis

## Abstract

**Highlights:**

**What are the main findings?**

- Technical specialties draw more medical complaints; communicative specialties draw more organizational complaints.
- Long-term management specialties show the highest share of organizational complaints (41.6%).

**What are the implications of the main findings?**

- PREMs can guide targeted quality improvement when analyzed by specialty-specific complaint patterns.
- Improving care in long-term specialties requires focusing on organizational processes, not just clinical skills.

**Abstract:**

**Background/Objectives:** Patient-Reported Experience Measures (PREMs) help us understand how patients perceive healthcare quality. Yet most studies look at complaints in isolation, without tying them to the structural features of medical practice. This study asks whether the nature of clinical work—shaped by diagnostic pathways, interaction patterns, and professional focus—predicts what patients complain about. **Methods:** We analyzed 18,492 negative reviews from infodoctor.ru, collected between 2012 and 2023 across 16 Russian cities with populations over one million. We used a mix of methods: machine learning (logistic regression) to classify complaints as medical (M-type) or organizational (O-type), statistical tests (chi-square, proportion analysis), and expert validation by nine independent specialists. We also built a novel multidimensional classification of medical practices based on three criteria: diagnostic pathway length, frequency and duration of patient interaction, and whether the work is mainly technical or communicative. **Results:** Technical specialties received far more medical complaints than communicative ones (39.8% vs. 29.3%, *p* < 0.001), while communicative specialties received more organizational complaints (45.7% vs. 35.0%, *p* < 0.001). Specialties that manage chronic conditions over the long term had the highest share of organizational complaints (41.6%). At the city level, the share of communicative specialists correlated negatively with complaints per capita (r = −0.541, *p* = 0.0306). We found no meaningful gender differences in complaint patterns. **Conclusions:** The type of medical practice systematically shapes what patients complain about. Technical specialties draw criticism on clinical quality; communicative specialties draw criticism on how care is organized. Long-term care faces challenges rooted more in administrative friction than in clinical competence. These findings show that PREMs, when analyzed through a practice-based lens, can support targeted quality improvement—moving from simply tracking complaints to acting on them in specialty-specific ways.

## 1. Introduction

The quality of healthcare services depends on many factors. A key distinction in quality assessment is between clinical (medical) and non-clinical (organizational, interaction-related) aspects [1,2]. Studies have consistently found that organizational elements—such as effective communication and patient comfort—often shape overall satisfaction just as much as clinical outcomes [3,4,5].

Patient-Reported Experience Measures (PREMs) have become essential tools for capturing how patients experience healthcare delivery [6,7]. Unlike Patient-Reported Outcome Measures (PROMs), which focus on clinical results, PREMs concentrate on the patient’s journey: access, communication, and the care environment. Understanding these experiences is critical for identifying where care can be improved.

The spread of online platforms where patients rate doctors and share their experiences has opened up a rich new source of data [8,9]. These platforms do more than reflect individual encounters—they also shape public perceptions of healthcare quality and represent a modern form of patient feedback [10,11]. Analyzing this data with advanced methods can reveal patterns that traditional surveys often miss.

To handle the large volumes of unstructured text from such platforms, researchers increasingly rely on machine learning and natural language processing (NLP) [12,13,14]. These techniques make it possible to extract themes and sentiments systematically. Yet, as a recent scoping review pointed out, there is still a noticeable gap between developing these analytical tools and actually using them to improve healthcare management [15]. The real challenge is turning analytical findings into actionable strategies.

Our earlier work laid the groundwork for tackling this challenge in the Russian context. We developed and tested a hybrid method to classify Russian-language patient reviews using neural network architectures (GRU, LSTM, CNN), achieving high accuracy for sentiment analysis (val_accuracy = 0.9271) [16,17]. That success allowed us to classify reviews using various criteria, including whether the complaint was aimed at a doctor or a clinic. We then built a comprehensive, open-access database of negative patient reviews from infodoctor.ru [18]. More recently, we developed and validated a machine learning algorithm to classify these reviews into two management-relevant categories: complaints about medical aspects (M-type) and complaints about organizational aspects (O-type) [19]. That analysis revealed significant variation in complaint patterns across cities and medical specialties.

At first glance, classifying reviews by whether they target a doctor or a clinic might seem like a straightforward way to separate medical from organizational complaints [16]. But this approach falls short because a large share of reviews are “mixed” (C-type): a doctor might be criticized for rudeness (an organizational issue), while the clinic is criticized for poor diagnostics (a medical issue). When we instead classified complaints by their actual medical or organizational content [19], we found stable patterns across specialties. For instance, patients of dermatologists, neurologists, and dentists complained more often about medical issues, while patients of gynecologists, urologists, and functional diagnostics specialists pointed more to organizational problems. These persistent differences led us to ask whether there is a deeper structure behind them.

Despite these advances, a critical gap remains. Our previous work [19] showed that complaint patterns differ by specialty, but it did not explain why. Are these differences random, or do they reflect deeper structural features of medical practice itself? Without an answer, quality improvement efforts risk being generic, applying the same fixes to dermatology and psychiatry, when their challenges may be fundamentally different.

This study addresses that gap by shifting the focus from specialties as discrete categories to the underlying dimensions of clinical work. We introduce a multidimensional classification based on three characteristics that define a medical practice: the length of the diagnostic pathway, the frequency and duration of patient interaction, and whether the work is predominantly technical or communicative. This approach lets us ask a novel question: does the nature of a medical practice systematically shape what patients complain about?

Specifically, we test three hypotheses: that specialties with a predominantly technical component receive more medical complaints, that specialties with a predominantly communicative component receive more organizational complaints, and that specialties involving long-term patient management receive the highest proportion of organizational complaints—reflecting accumulated administrative friction.

Building on this foundation, the present study adds a more nuanced analytical layer. We move beyond simply classifying complaints to understanding why they vary. Our central aim is to identify stable patterns linking the type of medical practice—defined by its diagnostic process, interaction pattern, and core professional activity—with the predominant type of patient complaint. In doing so, we hope to uncover structural challenges faced by different parts of the healthcare system and provide a basis for more targeted quality improvement.

## 2. Materials and Methods

### 2.1. Data Source

The study draws on Russian-language patient reviews collected from infodoctor.ru between July 2012 and August 2023. We chose this platform because it specializes in medical services, so the feedback is relevant and reasonably structured.

The inclusion criteria for the initial dataset were as follows:

- A rating of “1 star” out of 5, indicating a clearly negative review.
- The healthcare facility mentioned in the review is located in one of Russia’s 16 cities with a population exceeding one million.
- The review contains a textual description of the patient’s complaint.

The initial dataset was compiled in an earlier phase of our research [18] and includes 18,492 reviews with predefined characteristics: complaint type (M, O, or C); patient gender (determined through a combination of linguistic markers—grammatical gender endings like oбратилась/oбратился for “she/he addressed” and дoвoльна/дoвoлен for “satisfied”—and anthroponymic analysis of first names, patronyms, and last names such as Anna, Sergey, Ivanova, Petrov); geographical location; and the year of the review. This method identified gender with nearly 100% accuracy. The dataset is publicly available on Zenodo (10.5281/zenodo.15257447). During preprocessing, we removed duplicate entries and anonymized personal data in line with ethical standards.

For the current study, which focuses on complaints by medical practice, we created a subsample that includes only reviews clearly addressed to a named physician and containing enough information to identify the doctor’s specialty. After applying these criteria, the final analytical sample came to 9261 reviews.

### 2.2. Classification of Complaints

We classified complaint texts as medical (M-type) or organizational (O-type) using a combined approach. A review could be labeled M-type, O-type, or combined (C-type) if it contained elements of both.

Reviews from Moscow, three independent experts carried out manual coding. Inter-expert agreement was high: in 91.5% of cases, at least two of the three annotators assigned the same complaint type [18]. For reviews from other cities, we used a logistic regression model. The methodology is detailed in our previous work [19], where we reported classification accuracy of 88%.

### 2.3. Multidimensional Classification of Medical Specialties

To move beyond simply counting complaints and toward understanding their structural causes, we developed a three-dimensional classification of medical practices. The goal was to group specialties by characteristics of clinical work that might shape patient experience—and therefore the kinds of complaints patients make.

The classification was built in several stages. First, we used a large language model (DeepSeek, version V3.2) to generate initial ideas and organize information about typical diagnostic approaches, interaction patterns, and professional tasks across a wide range of specialties. The authors take full responsibility for the content and critically reviewed all AI-generated material. DeepSeek served only as an assistive tool to make the process more efficient; it did not replace our intellectual input in developing the research concept, interpreting results, or preparing the final manuscript.

Second—and crucially—this preliminary classification was reviewed, discussed, and validated by a panel of independent medical experts, including nine practicing physicians (Elena Vinogradova, endocrinologist and nutritionist, and eight others). This expert validation ensured the classification is content-valid and reflects current clinical practice. The final system thus combines AI-assisted analysis with professional medical expertise.

The complete classification for all 82 specialties is presented in Appendix A (Table A1). The three axes are defined below.

#### 2.3.1. Classification by the Length and Uncertainty of the Typical Diagnostic Pathway

This axis groups specialties by the typical journey from initial presentation to confirmed diagnosis. We focus on the pathway’s length and inherent uncertainty because these directly affect how many interactions patients have with the healthcare system—and thus potential points for dissatisfaction.

- Long diagnostic pathway: Initial symptoms are often non-specific and could point to several conditions. Arriving at an accurate diagnosis usually takes time, multiple consultations, and several tests. (Examples: neurology, gastroenterology, psychiatry, rheumatology).
- Medium diagnostic pathway: Symptoms tend to be more localized, and a diagnosis can often be reached based on those symptoms and standard examinations. (Examples: cardiology, gynecology, urology, dermatology, traumatology, dentistry, surgery).
- Short, technically determined pathway (diagnostic services): The visit is for a specific, well-defined procedure. The interaction is brief and not aimed at establishing a final clinical diagnosis. (Examples: ultrasound diagnostics, radiology, functional diagnostics).

Focusing on the length of the diagnostic pathway rather than its clinical complexity was a deliberate methodological choice. Expert consultation confirmed that classifying by “complexity” alone would be too simplistic, since most specialties use instrumental and laboratory methods to some degree. A patient-centered measure like pathway length better captures the patient’s accumulated experience and the points where dissatisfaction may arise—and it is also easier for patients themselves to perceive.

#### 2.3.2. Classification by the Frequency and Duration of Patient Interaction

This axis categorizes specialties by the typical pattern of interaction between doctor and patient, ranging from a single visit to long-term, ongoing care.

- Long-term management (multiple visits): Specialties focused on monitoring and managing chronic conditions, requiring regular follow-up over an extended period. (Examples: endocrinology, cardiology, rheumatology, general practice).
- Episodic interaction (limited number of visits): Specialties where patients typically seek care for a specific, often acute, problem that is resolved within a defined, limited number of visits. (Examples: surgery, cosmetology, dentistry).
- One-time visits (diagnostic services): Specialties where the service is provided within a single, self-contained diagnostic procedure. (Examples: ultrasound diagnostics, MRI).
- Mixed type: Specialties that combine long-term management of chronic patients with episodic consultations for acute issues. (Examples: general practice, gynecology, urology, otorhinolaryngology).

Medical experts validated this four-way classification, noting that a simple binary “long-term/episodic” scheme does not capture the full diversity of clinical practice. Diagnostic services were identified as a distinct category of interaction, warranting their own “one-time visits” label.

#### 2.3.3. Classification by the Predominant Component of Professional Activity

This axis groups specialties by the dominant skill set most critical to successful service delivery, while acknowledging that all medical practice combines these elements in varying proportions.

- Predominantly “technical” component: Specialties where outcomes—and how patients evaluate them—depend heavily on manual skills, instruments, and technical equipment. (Examples: surgery, dentistry, ultrasound diagnostics).
- Predominantly “communicative” component: Specialties where success depends critically on communication skills: taking a history, explaining, building rapport, and managing expectations. (Examples: psychiatry, psychotherapy, general practice, pediatrics).
- Mixed type: Specialties that require a balanced combination of advanced technical skills (including interpreting investigations) and well-developed communicative abilities. (Examples: oncology, gynecology, neurology).

Classifying specialties as “technical” or “communicative” is a simplification for analytical purposes. Expert validation confirmed that while most clinical practices ideally combine both elements, this distinction helps reveal structural differences in what patients expect. Diagnostic specialties (like ultrasound) are predominantly technical, while clinical specialties (like neurology) need a balance of both. The “mixed” category reflects fields where both components are essential; in an ideal world, every physician would belong here. But for this analysis, identifying a predominant component is necessary to uncover structural patterns.

### 2.4. Statistical Analysis

To test for associations between specialty groups and complaint types, we used Pearson’s chi-square test for independence. We also analyzed standardized residuals to identify which cells contributed most to significant results.

For the city-level analysis, we calculated Pearson correlation coefficients to explore the relationship between the proportion of communicative specialties in a city and the total number of complaints. We normalized complaint volume by city population to make comparisons fair.

All statistical analyses were carried out in Python (version 3.9) using the SciPy (version 1.10.1) and StatsModels (version 0.13.5) libraries. Python is available at https://www.python.org/ (accessed on 9 April 2026). We considered a *p*-value of less than 0.05 statistically significant.

## 3. Results

As defined in Section 2.2, M-type (medical) complaints refer to aspects of diagnosis and treatment effectiveness. O-type (organizational) complaints refer to staff interaction, waiting times, and the clinic environment. C-type (combined) complaints contain elements of both.

### 3.1. Association Between Predominant Professional Activity and Complaint Type

We found a significant association between a specialty’s predominant component (technical, communicative, or mixed) and the type of complaint it received (medical, organizational, or combined), χ^2^ (4, N = 9162) = 73.5, *p* < 0.001. Table 1 shows the distribution.

A post hoc analysis of standardized residuals showed that specialties with a predominantly technical component were significantly more likely than expected to receive medical complaints. In contrast, specialties with a predominantly communicative component were significantly more likely to receive organizational complaints. The mixed group fell closer to the overall average, with a slight tendency toward combined complaints.

### 3.2. Association Between Duration of Patient Interaction and Complaint Type

There was also a significant association between the typical duration of patient interaction and the type of complaint, χ^2^ (6, N = 9162) = 76.8, *p* < 0.001. Table 2 presents the distribution.

Specialties involving long-term patient management had the highest proportion of organizational complaints (41.6%). In contrast, specialties with episodic interaction had the highest proportion of medical complaints (42.4%). The pattern for one-time diagnostic visits was closer to that of the mixed type, suggesting that even though these visits are brief, patients may perceive them as part of a longer diagnostic process—making this group worth exploring further.

### 3.3. Association Between Diagnostic Pathway Length and Complaint Type

We also found a significant association between the length of the typical diagnostic pathway and the type of complaint, χ^2^ (4, N = 9162) = 25.0, *p* < 0.001. Table 3 shows the distribution.

Specialties with a long diagnostic pathway had the highest proportion of organizational complaints (40.8%). This supports the idea that when a condition requires a complex, prolonged diagnostic journey, organizational hurdles—scheduling, waiting, coordinating between specialists—can become a primary source of dissatisfaction, sometimes overshadowing concerns about the final medical outcome. In these specialties, the underlying problem often lies less in the physician’s competence and more in inefficient patient routing and organizational barriers.

### 3.4. City-Level Analysis

To explore possible systemic factors, we analyzed complaint patterns across the 16 cities. A Pearson correlation test showed a significant negative correlation between the proportion of communicative specialists in a city and the total number of complaints, normalized by population (r = −0.541, *p* = 0.0306). In other words, cities with a higher share of communicative specialists—such as general practitioners, psychiatrists, and pediatricians—tend to have fewer complaints per capita. Table 4 summarizes key city-level indicators.

The relationship between the share of long-term management specialties and complaint volume was also negative but did not reach statistical significance (r = −0.441, *p* = 0.0875).

### 3.5. Gender Differences

We found no statistically significant differences in complaint patterns by patient gender. The distribution of M-, O-, and C-type complaints was similar for men and women, both overall and within each of the three main specialty groups (technical, communicative, mixed), with all *p*-values > 0.05. This suggests that the associations we identified are robust across these demographic groups.

## 4. Discussion

### 4.1. Main Findings in Context

Our findings confirm that complaint patterns follow from the structural nature of medical practice. That aligns with a growing body of research linking practice characteristics to patient feedback. A recent systematic review of surgical PREMs by Darwish et al. [20] identified communication, waiting time, and coordination of care as key themes in surgical patient experience—echoing our observation that technical specialties attract complaints about process-related issues alongside clinical outcomes. More broadly, Friedel et al. [21] noted that cross-country comparisons of PREMs remain challenging because measurement tools vary, yet patient expectations consistently differ by care setting. Our work complements these studies by showing that such differences are not random but stem from the structural nature of medical practice.

A key finding is the clear divide between specialties with a predominantly technical focus and those with a predominantly communicative focus. Patients evaluating a “technical” service—like surgery or a diagnostic test—tend to focus their criticism on the quality and outcome of that technical intervention. In contrast, for services where the interaction itself is central—like therapy or psychiatry—dissatisfaction centers on communication, comfort, and organization. This suggests that quality improvement strategies need to be tailored to the core nature of the service.

More recent evidence reinforces this distinction. In a mixed-methods study from Ghana, Abdulai et al. [22] found that communication competence and interpersonal relationships had a greater impact on patient satisfaction than technical skills alone—technical skills contributed only a modest increase, while effective communication made a substantial difference. That supports our observation that in communicative specialties, dissatisfaction centers on the process of interaction, whereas in technical specialties, patients focus on clinical outcomes.

Our study also highlights a significant challenge for specialties involving long-term patient management, such as endocrinology and cardiology. These fields, which care for patients with chronic conditions, received the highest proportion of organizational complaints (41.6%). This suggests that for patients who must navigate the healthcare system repeatedly, the accumulated friction of scheduling appointments, waiting, and obtaining referrals can become a greater source of frustration than any single medical event. The challenge, then, lies not only in individual physician competence but also in the design of care pathways and the administrative processes that surround them.

The concept of administrative burden as a distinct driver of dissatisfaction has gained empirical traction. In a comprehensive scoping review, Kyle et al. [23] synthesized evidence on patient administrative burden—the non-clinical work patients must do to use the healthcare system—and found that tasks such as scheduling, referrals, and insurance navigation are consistently linked to frustration, especially among patients with complex or chronic conditions. Our findings extend this observation: specialties defined by long-term patient management receive the highest proportion of organizational complaints, suggesting that administrative friction builds up with each repeated interaction. Supporting this, Albaqami and Alshagrawi [24] reported that among patients with chronic diseases attending primary care in Saudi Arabia, dissatisfaction was most pronounced around access to care, coordination, and continuity—organizational factors that overshadowed clinical concerns. Together, these studies suggest that improving patient experience in chronic care requires investing in care coordination and administrative streamlining as much as—or more than—clinical training.

The city-level analysis adds another dimension. The negative correlation between the proportion of communicative specialists—such as general practitioners, psychiatrists, and pediatricians—and the overall volume of complaints suggests a protective role for a well-developed primary care sector. A strong primary care system may act as a filter, managing patient expectations and resolving issues early, thereby reducing the likelihood that patients turn to public online complaints. This observation, however, is correlational and needs further investigation to establish causality.

The evolving role of primary care physicians may help explain this protective effect. Bi and Liu [25] trace how UK general practitioners have shifted from passive “gatekeepers”—simply managing referrals—to proactive “health agents” who engage in health monitoring, patient education, and care coordination. This expanded role inherently involves communicative skills and proactive management of patient expectations. Our finding that a higher proportion of such specialists correlates with fewer complaints suggests that strengthening primary care systems may yield systemic benefits not only for clinical outcomes but also for patient experience.

### 4.2. What This Means for Practice

Our findings support shifting from generic quality improvement to targeted interventions tailored to practice characteristics.

For technical specialties (surgery, dentistry, diagnostic services), patient expectations center on clinical precision. Quality improvement should focus on adherence to technical protocols, clear communication about procedural risks, and post-procedure follow-up. Training programs in these fields might emphasize risk communication alongside technical skills.

For communicative specialties (psychiatry, general practice, pediatrics), patients are more sensitive to how they are treated. Interventions should target communication skills, shared decision-making, and efficient yet empathetic consultations. Our city-level finding suggests that investing in primary care—where communicative skills are paramount—could reduce patient frustration across the system.

For long-term management specialties, the priority should be reducing administrative friction. That means simplifying appointment scheduling, cutting wait times for routine follow-ups, improving referral coordination, and using digital tools to streamline patient-provider communication. These are not “soft” issues; they directly shape the experience of patients with chronic conditions who must navigate the system repeatedly.

### 4.3. Sociological and Methodological Implications

From a sociological perspective, this research shows how online platforms make the collective experience of millions of patients visible. The complaint patterns we identified reflect not only specific problems in physicians’ work but also broader shifts in public expectations: a growing demand for respectful communication, less tolerance for organizational complexity—especially during long-term treatment—and different expectations for technical versus relational services.

The absence of gender differences in complaint patterns is notable. It suggests that the structural drivers of dissatisfaction we identified—rooted in the type of medical practice itself—are strong enough to override any demographic differences in communication styles or expectations.

By linking specific, actionable practice characteristics to distinct profiles of patient feedback, this research provides a framework for moving from reactive complaint management to proactive, targeted quality improvement. The approach directly addresses the gap noted in the literature [15], offering a substantive analytical model that connects NLP-derived insights with traditional healthcare quality metrics and clinical practice.

### 4.4. Limitations

Several limitations should be noted. First, the data come from a single online platform (infodoctor.ru) and are limited to major Russian cities; findings may not generalize to rural settings or other countries with different healthcare structures. Second, platform users may overrepresent younger, more digitally active patients, potentially biasing the complaint landscape. Third, while our machine learning model (logistic regression) achieved 88% accuracy—which is robust for this task—modern transformer-based architectures offer potential for more nuanced text analysis. A recent scoping review by Cho et al. [26] found that models such as BioBERT and ClinicalBERT consistently outperform traditional classifiers on clinical text tasks, especially for capturing context-dependent linguistic patterns. Fourth, we could not reliably infer patient age from anonymous reviews, which limits demographic analysis. Fifth, the city-level findings are correlational and do not establish causality. Sixth, we did not conduct a formal post hoc power analysis; the sample size was determined by the availability of reviews meeting the inclusion criteria. Future research should aim to validate these findings across multiple platforms and settings, and test whether interventions targeting practice-specific complaint profiles actually improve patient experience.

## 5. Conclusions

This study shows that the type of medical practice systematically shapes what patients complain about. By building a multidimensional classification of medical practices—based on diagnostic pathway length, how often and how long patients interact with doctors, and whether the work is mainly technical or communicative—we found that technical specialties draw medical complaints, communicative specialties draw organizational complaints, and long-term management specialties have the highest share of organizational complaints (41.6%). These patterns held across cities and did not differ by gender.

Our findings shift the focus from simply tracking complaints to acting on them in ways that match each specialty. For technical fields, interventions should emphasize clinical precision and clear communication about risks. For communicative fields, training should focus on communication skills and shared decision-making. For long-term management, the priority is reducing administrative friction—scheduling, referrals, and care coordination.

Future research should validate these results across other platforms and settings, explore transformer-based NLP models for more detailed complaint analysis, and test whether interventions tailored to practice-specific complaint profiles actually improve patient experience.

## Figures and Tables

**Table 1:** Distribution of complaint types by predominant professional activity of the specialty.

Predominant Activity	Medical Complaints (%)	Organizational Complaints (%)	Combined Complaints (%)	Total (n)
Technical	39.8	35.0	25.2	3148
Communicative	29.3	45.7	25.1	1141
Mixed	33.3	37.6	29.1	4873

**Table 2:** Distribution of complaint types by the typical duration of patient interaction.

Interaction Type	Medical Complaints (%)	Organizational Complaints (%)	Combined Complaints (%)	Total (n)
Long-term management	31.8	41.6	26.6	3187
Episodic interaction	42.4	32.0	25.6	1938
One-time visits (diagnostic)	34.0	38.7	27.3	733
Mixed type	34.1	37.1	28.8	3304

**Table 3:** Distribution of complaint types by the length of the typical diagnostic pathway.

Diagnostic	Medical Complaints (%)	Organizational Complaints (%)	Combined Complaints (%)	Total (n)
Long diagnostic pathway	31.5	40.8	27.7	2642
Medium diagnostic pathway	36.8	36.2	27.0	5787
Short, technically determined	34.0	38.7	27.3	733

**Table 4:** Distribution of key indicators across cities with populations over one million.

City	Medical Complaints (%)	Share of Communicative Specialties (%)	Share of Long-Term Management Specialties (%)	Complaints Per 1 Million Population
Moscow	33.2	10.1	30.9	384.4
Krasnodar	31.2	18.3	41.4	319.8
Nizhny Novgorod	32.2	16.2	44.0	317.5
Voronezh	32.9	13.6	35.8	231.0
Rostov-on-Don	26.9	14.2	37.9	222.6
St. Petersburg	35.6	12.1	34.0	219.9
Yekaterinburg	28.7	19.7	42.5	209.7
Samara	37.3	19.3	43.0	198.9
Kazan	33.2	11.8	31.4	182.0
Chelyabinsk	30.6	16.6	47.7	162.8
Ufa	36.5	13.8	34.8	160.4
Novosibirsk	34.7	20.7	52.1	149.2
Krasnoyarsk	31.7	19.0	50.0	129.3
Volgograd	37.7	15.6	34.4	121.4
Perm	28.2	15.4	42.7	111.7
Omsk	36.6	20.5	47.3	98.0

## Data Availability

The raw data supporting the conclusions of this article were derived from the publicly available source infodoctor.ru under its terms of service. The processed dataset, including the 9261-review annotated corpus used for analysis in this study, is publicly available on Zenodo (10.5281/zenodo.15257447). The code used for analysis and the full classification manual (Appendix A) are available from the corresponding author upon reasonable request.

## Abbreviations

The following abbreviations are used in this manuscript:

PREMs	Patient-Reported Experience Measures
PROMs	Patient-Reported Outcome Measures
PRMs	Patient-Reported Measures
M-type	Medical complaint type
O-type	Organizational complaint type
C-type	Combined complaint type
NLP	Natural Language Processing

## Appendix A

This appendix presents the full multidimensional classification for the 82 medical specialties in the study. The classification was developed in several stages, combining AI-assisted analysis with validation by a panel of independent medical experts. For each specialty, the table shows the category assignment for all three axes, along with a brief justification.

**Table A1:** Complete classification of medical specialties with justifications.

Specialty	Diagnostic Pathway Length	Patient Interaction Type	Predominant Professional Activity Component
Abdominal surgeon	Medium diagnostic pathway (Problems often localized, diagnosis via ultrasound/CT)	Episodic interaction (Surgical intervention, limited contact)	Technical (Surgical skills, operative technique)
Allergist	Long diagnostic pathway (Complex differential diagnosis, cross-reactions)	Long-term management (Chronic conditions, seasonal monitoring)	Mixed (Test interpretation + explanation of treatment regimens)
Andrologist	Medium diagnostic pathway (Specific diagnostic methods, but localized area)	Mixed type (Long-term treatment + one-time procedures)	Mixed (Procedural skills + sensitive conversations)
Anesthesiologist	Medium diagnostic pathway (Assessment by standard protocols, monitoring)	Episodic interaction (Perioperative interaction)	Technical (Anesthesia management, resuscitation skills)
Audiologist	Medium diagnostic pathway (Audiometric diagnostics)	Long-term management (Selection and adjustment of hearing aids)	Mixed (Diagnosis + rehabilitation)
Cardiac surgeon	Medium diagnostic pathway (Preoperative diagnosis, surgical profile)	Episodic interaction (Surgical treatment)	Technical (Surgical skills)
Cardiologist	Medium diagnostic pathway (Standardized methods, but complex cases of arrhythmias, ischemic heart disease)	Long-term management (Chronic heart diseases)	Mixed (ECG/Holter interpretation + patient management)
Coloproctologist	Medium diagnostic pathway (Specific diagnostic methods)	Mixed type (Chronic diseases + surgery)	Mixed (Endoscopy + surgery)
Cosmetologist	Medium diagnostic pathway (Aesthetic assessment, standard protocols)	Episodic interaction (Cosmetic procedures)	Technical (Performing procedures)
CT specialist	Short, technically determined pathway (Conducting and describing studies)	One-time visits (Diagnostic service)	Technical (Work with equipment)
Defectologist	Medium diagnostic pathway (Assessment by standardized methods)	Long-term management (Long-term correction courses)	Communicative (Work with speech, communication)
Dental surgeon	Medium diagnostic pathway (Preoperative diagnostics)	Episodic interaction (Surgical treatment)	Technical (Surgical skills)
Dentist	Medium diagnostic pathway (Visual diagnostics, X-ray)	Episodic interaction (Dental treatment)	Technical (Dental procedures)
Dermatologist	Medium diagnostic pathway (Often visual diagnosis, but complex differential cases)	Mixed type (Chronic diseases + one-time consultations)	Mixed (Visual diagnosis + explanation)
Diabetologist	Long diagnostic pathway (Complex endocrine pathology, therapy adjustment)	Long-term management (Lifelong monitoring)	Mixed (Treatment adjustment + patient education)
Endocrinologist	Long diagnostic pathway (Complex hormonal disorders, differential diagnosis)	Long-term management (Chronic diseases)	Mixed (Complex diagnostics + treatment management)
Endoscopist	Short, technically determined pathway (Conducting endoscopic examinations)	One-time visits (Diagnostic service)	Technical (Proficiency in endoscopic techniques)
Epileptologist	Long diagnostic pathway (Complex neurological diagnosis of paroxysmal conditions)	Long-term management (Long-term treatment)	Mixed (Complex diagnostics + therapy selection)
Exercise therapy instructor	Medium diagnostic pathway (Assessment by functional tests)	Long-term management (Exercise courses)	Technical (Demonstration, exercise correction)
Functional diagnostics specialist	Short, technically determined pathway (Conducting specialized studies)	One-time visits (Diagnostic service)	Technical (Operation of complex equipment)
Gastroenterologist	Long diagnostic pathway (Non-specific symptoms, differential diagnosis)	Long-term management (Chronic gastrointestinal diseases)	Mixed (Endoscopy + management of chronic patients)
General practitioner	Long diagnostic pathway (Wide range of diseases, primary diagnosis)	Long-term management (Ongoing patient monitoring)	Communicative (History taking, treatment coordination)
Geneticist	Long diagnostic pathway (Specialized laboratory diagnostics and interpretation of complex genetic data)	One-time visits (Consultation + analysis)	Mixed (Complex data interpretation + counseling)
Gynecologist	Medium diagnostic pathway (Combination of examination, ultrasound, tests)	Mixed type (Regular check-ups + acute conditions)	Mixed (Procedural skills + sensitive communication)
Gynecologist-endocrinologist	Long diagnostic pathway (Complex hormonal disorders, differential diagnosis)	Long-term management (Long-term hormonal correction)	Mixed (Complex diagnostics + treatment management)
Hematologist	Long diagnostic pathway (Complex laboratory diagnostics, rare diseases)	Long-term management (Long-term treatment, chemotherapy)	Mixed (Test interpretation + treatment management)
Hepatologist	Long diagnostic pathway (Specialization within gastroenterology, complex cases)	Long-term management (Chronic liver diseases)	Mixed (Complex diagnostics + long-term monitoring)
Immunologist	Long diagnostic pathway (Complex immunological disorders, rare diseases)	Long-term management (Chronic immune disorders)	Mixed (Complex diagnostics + treatment management)
Implantologist	Medium diagnostic pathway (CT planning, surgical protocols)	Episodic interaction (Surgical intervention)	Technical (Surgical skills)
Infectious disease specialist	Long diagnostic pathway (Complex differential diagnosis, epidemiological history)	Mixed type (Acute conditions + follow-up monitoring)	Mixed (Diagnosis + management of infectious patients)
Laboratory diagnostics specialist	Short, technically determined pathway (Interpretation of tests without clinical examination)	One-time visits (Diagnostic service provision)	Technical (Work with equipment, methodologies)
Laser therapist	Medium diagnostic pathway (Performing procedures based on indications)	Episodic interaction (Courses of procedures)	Technical (Work with equipment)
Mammologist	Medium diagnostic pathway (Combination of examination, ultrasound, mammography)	Mixed type (Regular check-ups + acute cases)	Mixed (Diagnosis + patient management)
Manual therapist	Medium diagnostic pathway (Diagnosis by manual methods)	Episodic interaction (Treatment sessions)	Technical (Manual techniques)
Massage therapist	Medium diagnostic pathway (Assessment of muscle tone)	Episodic interaction (Massage sessions)	Technical (Massage techniques)
Maxillofacial surgeon	Medium diagnostic pathway (Preoperative diagnostics)	Episodic interaction (Surgical treatment)	Technical (Surgical skills)
MRI specialist	Short, technically determined pathway (Conducting and describing studies)	One-time visits (Diagnostic service)	Technical (Work with complex equipment)
Mycologist	Medium diagnostic pathway (Laboratory diagnostics of fungal diseases)	Mixed type (Long-term treatment + consultations)	Mixed (Diagnosis + treatment management)
Narcologist	Long diagnostic pathway (Psychosomatic aspects, differential diagnosis)	Long-term management (Long-term rehabilitation)	Communicative (Psychotherapeutic work)
Neonatologist	Long diagnostic pathway (Characteristics of newborn period, non-specific symptoms)	Long-term management (Monitoring in newborn period)	Mixed (Diagnosis + newborn care)
Nephrologist	Long diagnostic pathway (Complex kidney pathology, differential diagnosis)	Long-term management (Chronic kidney diseases)	Mixed (Diagnosis + management of chronic patients)
Neurologist	Long diagnostic pathway (Complex neurological status, differential diagnosis)	Long-term management (Chronic diseases)	Mixed (Neurological examination + patient management)
Neuropsychologist	Long diagnostic pathway (Complex psychological diagnostics)	Long-term management (Long-term correction)	Communicative (Psychological diagnostics and correction)
Neurosurgeon	Medium diagnostic pathway (Preoperative diagnosis, surgical profile)	Episodic interaction (Surgical treatment)	Technical (Surgical skills)
Nutritionist	Medium diagnostic pathway (Analysis of food diary, biochemical parameters)	Long-term management (Long-term support)	Communicative (Counseling, motivation)
Obstetrician	Medium diagnostic pathway (Combination of visual examination, ultrasound, lab parameters)	Mixed type (Long-term pregnancy management + one-time delivery)	Mixed (Technical skills in delivery + communication)
Occupational pathologist	Medium diagnostic pathway (Link between diseases and occupational activity)	Long-term management (Regular check-ups)	Mixed (Expertise + counseling)
Oncologist	Medium diagnostic pathway (Histological verification, but complex treatment choices)	Long-term management (Long-term treatment and monitoring)	Mixed (Treatment choice + patient management)
Ophthalmic surgeon	Medium diagnostic pathway (Preoperative diagnostics)	Episodic interaction (Surgical treatment)	Technical (Microsurgical skills)
Ophthalmologist	Medium diagnostic pathway (Specialized equipment, but standardized methods)	Mixed type (Regular check-ups + acute conditions)	Mixed (Equipment-based diagnosis + treatment)
Orthodontist	Medium diagnostic pathway (Diagnosis by CT, models)	Long-term management (Long-term orthodontic treatment)	Technical (Planning and correction of appliances)
Orthopedist	Medium diagnostic pathway (Musculoskeletal diagnostics, radiology)	Mixed type (Chronic conditions + injuries)	Technical (Correction, prosthetics)
Otorhinolaryngologist	Medium diagnostic pathway (Combination of examination, endoscopy, audiometry)	Mixed type (Acute conditions + chronic diseases)	Mixed (Diagnostic endoscopy + surgery + conservative treatment)
Pediatrician	Long diagnostic pathway (Characteristics of childhood, non-specific symptoms)	Long-term management (Monitoring from birth)	Communicative (Work with children and parents)
Periodontist	Medium diagnostic pathway (Diagnosis of periodontal diseases)	Long-term management (Long-term treatment)	Technical (Dental procedures)
Phlebologist	Medium diagnostic pathway (Ultrasound diagnostics of venous system)	Mixed type (Chronic diseases + acute conditions)	Mixed (Diagnosis + minimally invasive procedures)
Phthisiatrician	Long diagnostic pathway (Complex differential diagnosis of tuberculosis)	Long-term management (Long-term treatment)	Mixed (Diagnosis + treatment management)
Physiotherapist	Medium diagnostic pathway (Prescription based on indications from treating physician)	Long-term management (Courses of physiotherapy)	Technical (Operation of physiotherapy equipment)
Plastic surgeon	Medium diagnostic pathway (Aesthetic assessment, surgical planning)	Episodic interaction (Surgical treatment)	Technical (Surgical skills)
Podologist	Medium diagnostic pathway (Foot diagnostics)	Episodic interaction (Care procedures)	Technical (Performing procedures)
Psychiatrist	Long diagnostic pathway (Complex psychiatric diagnostics, differential diagnosis)	Long-term management (Long-term treatment)	Communicative (Psychiatric examination, psychotherapy)
Psychotherapist	Long diagnostic pathway (In-depth psychological diagnostics)	Long-term management (Long-term psychotherapy)	Communicative (Psychotherapeutic techniques)
Pulmonologist	Medium diagnostic pathway (Specific diagnostic methods for lung diseases)	Long-term management (Chronic diseases)	Mixed (Diagnosis + patient management)
Radiologist	Short, technically determined pathway (Interpretation of images)	One-time visits (Diagnostic service)	Technical (Work with equipment, image analysis)
Reproductologist	Medium diagnostic pathway (Complex diagnostic search for infertility causes)	Long-term management (Long-term IVF programs)	Mixed (Complex procedures + management of couples)
Rheumatologist	Long diagnostic pathway (Complex systemic diseases, autoimmune processes)	Long-term management (Chronic diseases)	Mixed (Complex diagnostics + long-term treatment)
Sexologist	Long diagnostic pathway (Complex psychosomatic aspects)	Long-term management (Long-term correction)	Communicative (Psychological counseling)
Somnologist	Long diagnostic pathway (Complex diagnostics of sleep disorders)	Long-term management (Long-term correction)	Mixed (Diagnosis + correction of disorders)
Speech therapist	Medium diagnostic pathway (Diagnosis by standardized methods)	Long-term management (Long-term correction courses)	Communicative (Work with speech)
Sports medicine physician	Medium diagnostic pathway (Specific sports injuries and conditions)	Mixed type (Regular check-ups + acute cases)	Mixed (Diagnosis + recommendations on physical activity)
Surgeon	Medium diagnostic pathway (Preoperative diagnostics)	Episodic interaction (Surgical treatment)	Technical (Surgical skills)
Therapist	Long diagnostic pathway (Wide range of diseases, primary diagnosis)	Long-term management (Ongoing monitoring)	Communicative (History taking, coordination)
Thoracic surgeon	Medium diagnostic pathway (Preoperative diagnostics)	Episodic interaction (Surgical treatment)	Technical (Surgical skills)
Toxicologist	Long diagnostic pathway (Determination of toxin type, dose, mechanism of action often with absent history)	Mixed type (Emergency care for acute poisoning + long-term management of chronic intoxications)	Mixed (Technical skills of detoxification + communication with patient and relatives to clarify circumstances)
Transfusiologist	Long diagnostic pathway (Complex immunohematological diagnostics)	Long-term management (Management of patients needing transfusions)	Technical (Work with blood components)
Traumatologist	Medium diagnostic pathway (X-ray diagnostics of injuries)	Mixed type (Acute injuries + rehabilitation)	Technical (Reduction, immobilization)
Trichologist	Medium diagnostic pathway (Comprehensive diagnosis of hair loss causes)	Mixed type (Long-term treatment + consultations)	Mixed (Diagnosis + treatment prescription)
Ultrasound diagnostician	Short, technically determined pathway (Conducting ultrasound examinations)	One-time visits (Diagnostic service)	Technical (Operation of equipment and image interpretation)
Urologist	Medium diagnostic pathway (Specific diagnostic methods)	Mixed type (Chronic diseases + acute conditions)	Mixed (Endoscopy + surgery + conservative treatment)
Vascular surgeon	Medium diagnostic pathway (Ultrasound diagnostics, angiography)	Episodic interaction (Surgical treatment)	Technical (Surgical skills)
Venereologist	Medium diagnostic pathway (Laboratory diagnostics, but often typical clinical picture)	Episodic interaction (Consultation for acute problem)	Technical (Sample collection, procedures)
Vertebrologist	Medium diagnostic pathway (Musculoskeletal problems, imaging)	Mixed type (Treatment courses + one-time consultations)	Mixed (Manual techniques + explanation)
